# Potential Therapeutic Effects of Neurotrophins for Acute and Chronic Neurological Diseases

**DOI:** 10.1155/2014/601084

**Published:** 2014-04-09

**Authors:** Junying Cai, Fuzhou Hua, Linhui Yuan, Wei Tang, Jun Lu, Shuchun Yu, Xifeng Wang, Yanhui Hu

**Affiliations:** ^1^Department of Anesthesiology, The Second Affiliated Hospital of Nanchang University, Nanchang, Jiangxi 330006, China; ^2^Department of Urology, The Second Affiliated Hospital of Nanchang University, Nanchang, Jiangxi 330006, China; ^3^Department of Anesthesiology, The first Affiliated Hospital of Nanchang University, Nanchang, Jiangxi 330006, China

## Abstract

The neurotrophins (NTs) nerve growth factor (NGF), brain-derived neurotrophic factor (BDNF), NT-3, and NT-4/5 are proteins that regulate cell proliferation, differentiation, and survival in both the developing and mature central nervous system (CNS) by binding to two receptor classes, Trk receptors and p75 NTR. Motivated by the broad growth- and survival-promoting effects of these proteins, numerous studies have attempted to use exogenous NTs to prevent the death of cells that are associated with neurological disease or promote the regeneration of severed axons caused by mechanical injury. Indeed, such neurotrophic effects have been repeatedly demonstrated in animal models of stroke, nerve injury, and neurodegenerative disease. However, limitations, including the short biological half-lives and poor blood-brain permeability of these proteins, prevent routine application from treating human disease. In this report, we reviewed evidence for the neuroprotective efficacy of NTs in animal models, highlighting outstanding technical challenges and discussing more recent attempts to harness the neuroprotective capacity of endogenous NTs using small molecule inducers and cell transplantation.

## 1. Introduction: Current Challenges in the Clinical Use of Neurotrophins

Preclinical research over the past 3 decades has described in immense detail the signaling pathways that lead to neuronal cell death [1, 2]. Moreover, a plethora of neuroprotective strategies have been developed for ameliorating brain damage and preserving or restoring neurological function in animal models of stroke, Alzheimer's disease (AD), Parkinson's disease (PD), Huntington's disease (HD), and other neurological disorders [3–5]. Such treatments include the administration of neurotrophic factors, which are endogenous proteins critical for proliferation, differentiation, and survival during development and neuroplasticity throughout life [6, 7]. Indeed, brain delivery of exogenous neurotrophic factors, such as the neurotrophin nerve growth factor (NGF) and brain-derived neurotrophic factor (BDNF), can reduce infarct volumes by 60–90% with near total restoration of behavioral function in experimental stroke models.

Despite this success in the laboratory, there are currently no broadly effective treatments to reverse or halt the progression of these diseases in patients.

Numerous theories have been advanced to explain the failure of neurotrophins in clinical trials, including limited access of peripherally administered neurotrophins to the central nerve system (CNS), the short biological half-lives of neurotrophins, the multimodal nature of disease progression, and the relatively short temporal window in which such treatments are effective (at least for acute neural insults such as stroke) [8]. For example, antistroke therapies must be instituted within 1–6 h of the event for significant efficacy, and few stroke patients can be treated within this timeframe. Thus, prophylactic strategies that upregulate endogenous protective capacity may be needed.

In recent years, methods for delivery of neurotrophins across the blood brain barrier (BBB) have advanced rapidly, as has the development of smaller neurotrophin receptor (Trk) agonists with significantly longer biological half-lives and BBB permeability than native neurotrophins like BDNF (serum half-life of ≈10 min) [9]. Perhaps the most difficult problem is that of delivering these molecules to appropriate targets, such as astrocyte cells, microglia cells, and vascular endothelial cells as well as neurons, while limiting exposure to undamaged brain tissue.

Despite these challenges, we may be close to developing neurotrophin receptor agonists and delivery systems that allow for rapid brain penetration to protect vulnerable human neurons from cell death. This goal may be facilitated by the fact that the many distinct pathogenic processes associated with stroke, trauma, and neurodegenerative diseases appear to converge on several mutually reinforcing final common pathways: excitotoxicity by excess glutamate receptor stimulation, intracellular calcium overload, oxidative stress, mitochondrial failure, and apoptosis. Here, we review evidence that neurotrophic factors can prevent neuronal death in acute and chronic brain diseases and highlight the remaining problems that have thus far prevented clinical application.

## 2. Neurotrophin-Mediated Protection against Acute Neural Insult

### 2.1. Direct Neurotrophin Administration Reduces Stroke Damage

Numerous studies have demonstrated that neurotrophins, particularly BDNF, reduce infarct volume in rodents when administered before, during, and/or after experimental stroke [10]. These studies have used various strategies to enhance the level of BDNF in brain. Intraventricular injection of BDNF for 8 days at the beginning of 24 hours before permanent middle cerebral artery occlusion (MCAO) in Wistar rats was found to improve neurological deficits [11]. Similarly, BDNF was delivered into the territory of the MCA by an osmotic minipump shortly after occlusion reduced cortical infarct volume by 37% [12]. Infusion of recombinant BDNF into Sprague-Dawley rat neocortex by an implanted osmotic minipump 2.5–14 days before, during, and for 2 days following MCAO reduced infarct volume as measured 2 days after stroke without affecting cerebral blood flow [13]. Continuous BDNF infusion immediately following 2 h right MCAO in rats reduced both cortical and subcortical infarct volume as well as the number of neurons in the ischemic penumbra expressing the proapoptotic factor Bax, while increasing the number of neurons expressing antiapoptotic Bcl-2 [14]. It was found that hippocampal CA1 pyramidal neurons were rescued by intraventricular injection of a viral vector encoding BDNF, as well as similar vectors encoding glial-derived neurotrophic factor (GDNF), NGF, insulin-like growth factor-1 (IGF-1), or vascular endothelial growth factor (VEGF) 30 min after the ischemic insult [15]. Transplantation of fresh bone marrow (BM) together with BDNF into the ischemic boundary zone (IBZ) of the rat brain following MCAO facilitated functional recovery of sensory and motor functions [16]. The effects of BDNF on infarct size were synergistic with brain hypothermia, another well-described antistroke treatment. Furthermore, postischemic striatal glutamate concentrations were reduced to a greater extent by combining BDNF and hypothermia than for either treatment alone [17].

Transplantation of choroid plexus (CP), which is known to secrete a variety of growth factors including BDNF, GDNF, and NGF, reduced infarct volume in rats [18]. Injection of neural progenitors into the hippocampus immediately after cerebral ischemia increased spatial memory performance in the Morris water maze (MWM) 12–28 days after cerebral ischemia and reduced infarct volume. Injection of neural progenitor cells (NPCs) also reversed the postischemic decline in BDNF expression [19]. Sodium orthovanadate protected cortical and hippocampal neurons after experimental subarachnoid hemorrhage by increasing BDNF and inhibiting BDNF receptor TrkB inactivation, effects that were abolished by pretreatment with the TrkB inhibitor K252a [20].

While most of these studies examined infarct formation or neurological recovery in the early period following ischemia, a more recent study demonstrated that transplantation of neural stem cells (NSCs) overexpressing BDNF improved neurological function as late as 12 weeks after MCAO [21]. In addition to grey matter damage, BDNF may also reduce damage caused by lacunar-type stroke in primates following occlusion of the deep subcortical penetrating arteries. Moreover, expression of BDNF was stimulated in the affected white matter following ischemic injury [22]. In a similar study, BDNF did not reduce infarct volume but still resulted in greater functional recovery following ischemia compared to controls. In addition, this treatment reduced astrogliosis, a process that may impede long-term recovery by blocking regrowth of axons [23].

Conversely, mice lacking a single BDNF allele or both neurotrophin-4 alleles (nt4^−^/^−^) exhibited larger cerebral infarcts than wild-type littermates [24]. Transgenic mice overexpressing the dominant-negative truncated variant of the BDNF receptor (TrkB.T1) specifically in cortical and hippocampal neurons exhibited greater damage in the cortex but not in regions (striatum) without TrkB.T1 transgene expression [25]. Infusion of an antisense BDNF oligonucleotide for 28 days after unilateral cortical ischemia blocked BDNF mRNA expression and negated the beneficial effects of motor rehabilitation on the recovery of skilled reaching by the contralateral limb but had no effect on motor function of the ipsilateral limb [26].

While neurotrophins are broadly neuroprotective against experimental stoke, several studies have shown greater efficacy of one particular neurotrophin over others as well as superior protection in specific brain regions. Infusion of BDNF into the substantia nigra (SN) had no effect on survival of nigrostriatal projection neurons after stroke and even exacerbated death of some striatal cholinergic and GABAergic interneurons [27]. Conversely, BDNF may be more effective than other neurotrophins against stroke-induced damage in the cortex. Mesenchymal stem cells (MSCs) overexpressing either BDNF or GDNF improved functional outcome as revealed by MRI one and two weeks after MCAO, while MSCs overexpressing the neurotrophin NT-3 did not [28]. Thus, region-specific administration may be necessary for full efficacy, and this constitutes a major hurdle for noninvasive peripheral administration.

### 2.2. Neurotrophins Mediate the Protective Efficacy of Other Treatments

The neuroprotective efficacies of many other manipulations that rescue neurons from stroke damage are mediated at least in part by enhanced expression of neurotrophins, particularly BDNF. For example, brief daily treadmill running for 3 weeks prior to stroke reduced infarct volumes in the frontoparietal cortex and dorsolateral striatum, and these effects were correlated with upregulation of NGF and BDNF in cortical neurons and striatal glia. In addition, there was a significant increase in microvessel density in the striatum [29]. The neuroprotective effects of fibroblast growth factor 2 (FGF-2) following MCAO may also be mediated in part by BDNF; FGF-2 null mice exhibited dramatically larger infarct volumes than wild-type littermates and a diminished postischemic induction of hippocampal BDNF and TrkB mRNA [30]. Cortical spreading depression (SD), which is a wave of depolarization followed by neuronal quiescence, can induce a preconditioning effect involving BDNF expression. Induced SD for 6 or 9 days prior to experimental focal ischemia reduced infarct volume and resulted in less severe neurological deficits both at one and 14 days after ischemia. This preconditioning effect was not observed in heterozygous BDNF-deficient mice [31]. Preconditioning can also be achieved by sublethal doses of N-methyl D-asparate (NMDA), which increased hippocampal CA1 neuron survival after ischemia, an effect blocked by the TrkB antagonist K252a [32]. Lithium is another widely studied neuroprotectant that requires BDNF/TrkB signaling [33]. Lithium-mediated neuroprotection involves GSK-3 inhibition, which in turn leads to enhanced BDNF expression [34]. Atorvastatin (Lipitor) treatment starting 24 h after MCAO also improved functional recovery and concomitantly increased BDNF expression (as well as VEGF and VEGFR2) at the ischemic border. In addition, atorvastatin treatment increased the number of migrating neurons, synaptophysin-positive cells, and angiogenesis around the infarct.* In vitro*, atorvastatin enhanced the migration of subventricular zone (SVZ) cells, an effect inhibited by a BDNF antibody [35].

In addition to invasive methods like cell therapy, brain concentrations of BDNF can be elevated by orally administered agents. The rennin-angiotensin system inhibitor candesartan was found to reduce stroke volume, improve neurological outcome, and promote TrkB expression [36]. Inhibitors of nitric oxide production may also be effective. The endothelial cell isoform of nitric oxide synthase (eNOS) protected against stroke through BDNF upregulation [37]; eNOS knockout mice exhibited more severe deficits than wild-type mice following permanent right MCAO, as well as reduced progenitor cell proliferation and migration from the SVZ and decreased angiogenesis at the ischemic boarder. These changes were associated with lower postischemic BDNF (but no change in VEGF and bFGF expression). Moreover, neurosphere formation and proliferation and neurite outgrowth were reduced in cultured SVZ cells derived from eNOS^−^/^−^ mice, characteristics reversed by BDNF administration [37]. Treadmill exercise prior to experimental ischemia improved motor performance and upregulated BDNF and TrkB expression in the contralateral hemisphere [38]. Significantly, enhanced BDNF only occurred following voluntary exercise, possibly because “forced” exercise enhances serum corticosterone, which may reduce neurotrophin expression [39]. Allosteric AMPAR modulators enhanced limb control when administered several days after stroke, but impaired recovery when administered immediately. Inhibition of BDNF function in the cortex preinfarct blocked this motor rescue [40].

Note, however, that other neuroprotective effects appear independently of neurotrophin expression. Preconditioning by sublethal MCAO 3 days prior to (normally) neurotoxic MCAO protected rats but did not alter expression of most neurotrophins, though BDNF expression was transiently reduced [41]. Housing rats in an enriched environment after experimental stroke was found to enhance recovery, but this was associated with a decrease in BDNF mRNA and protein expression, and enriched BDNF(+/−) mice showed improved motor function on the rotorod two weeks after transient MCAO compared to wild-type mice [42]. Enriched environments thus appear to enhance functional recovery after ischemic stroke independently of BDNF [43].

### 2.3. Novel Neurotrophin Delivery, Induction, and Targeting Methods

Several novel delivery systems may allow for noninvasive neurotrophin administration [44]. A single systemic administration of BDNF conjugated to the OX26 murine monoclonal antibody against the transferrin receptor, which facilitates transport across the BBB via the transferrin receptor transcytosis system, reduced infarct volume by 70% (although it had no effect on subcortical stroke volume) [45]. In another study, BDNF conjugated to a transferrin receptor antibody reduced infarct volume by 65–70% and improved rotorod performance [46]. High-voltage electric potential (HELP) stimulation of adult mice also enhanced BDNF levels, increased resistance to cerebral infarction, and improved escape latency in the MWM [47]. BDNF engineered to bind laminin, which is highly expressed in the ischemic brain, reduced infarct volume and improved neurological function as well as neurogenesis in the dentate gyrus [48]. Intranasal BDNF 2 h after reperfusion reduced the number of TUNEL-positive (apoptotic) neurons (but not infarct volume). This BDNF application also enhanced microglial activation (as indicated by OX-42 expression) and the number of phagocytotic microglia (ED1-positive), suppressed tumor necrosis factor-**α** expression, and enhanced expression of the anti-inflammatory cytokine interleukin-10 [49].

In addition to novel delivery/targeting systems for native BDNF, there have been extensive efforts to develop small mimetics with superior pharmacokinetic properties and BBB permeability [9]. For example, 7,8-dihydroxyflavone, a high-affinity TrkB agonist, was found to inhibit kainic acid-induced toxicity, decrease infarct volume following experimental stroke in a TrkB-dependent manner, and be neuroprotective in an animal model of Parkinson's disease [50].

### 2.4. Neuroprotection by Natural Neurotrophin Inducers

Several natural compounds have demonstrated neuroprotective efficacy mediated in part by BDNF upregulation. The plant flavonoids baicalin and jasminoidin in combination promoted BDNF expression and inhibited the apoptotic effector caspase-3 [51]. Niacin also increased BDNF and TrkB expression in neurons and reduced infarct volume* in vivo *following experimental stroke [52]. Infusion of galectin-1, an endogenous mammalian lectin, enhanced the expression and secretion of astrocytic BDNF, reduced the number of apoptotic neurons at the ischemic boundary, and improved functional recovery after experimental stroke [53]. p-Hydroxybenzyl alcohol (HBA), an active component of gastrodia elata blume, administered 1 h before MCAO, decreased infarct volume, promoted functional recovery as measured by the modified neurological severity score, and enhanced Nrf2, BDNF, GDNF, and MBP expression levels [54].

### 2.5. Mechanisms of Neurotrophin-Mediated Neuroprotection

Oxidative stress and intracellular-free calcium dysregulation are mutually reinforcing early events that lead to neuronal death following stroke [2]. Thus, much stroke research has focused on developing strategies to reduce free radical accumulation, augment cellular antioxidant defenses, chelate intracellular calcium, or interfere with upstream events such as excess glutamate receptor stimulation (excitotoxicity) in the ischemic brain [3]. There is an emerging consensus that inflammation is also critical in stroke pathogenesis [5], indicating that anti-inflammatory drugs are feasible therapeutic options. It is thus noteworthy that neurotrophins like BDNF can stabilize intracellular calcium, induce antioxidant enzyme expression, and suppress the release of proinflammatory cytokines [4, 55]. One mechanism of BDNF-induced protection relevant to both acute and chronic models of neurological disease is preservation of mitochondrial function. BDNF (but not GDNF or NGF) substantially increased the respiratory control index (RCI, a measure of respiratory coupling efficiency and ATP synthesis) in synaptosomes [56] but not in pure mitochondria nor in synaptosomes treated with an inhibitor of complex I, suggesting that activity of a TrkB-associated signaling pathway is necessary. Indeed, the effect was also blocked by inhibitors of the MEK-Bcl-2 pathway, the major pathway implicated in BDNF-TrkB-dependent neuroprotection [56].

Another possible mechanism for these neuroprotective effects is enhanced neurogenesis and migration of neural precursor cells (NPCs) from the SVZ. Daily intravenous bolus applications of BDNF after stroke resulted in significantly improved sensorimotor scores 6 weeks after stoke as well as enhanced neurogenesis in the DG and SVZ [57]. Continuous intraventricular BDNF infusion for two weeks enhanced the number of proliferating (doublecortin-positive) SVZ cells surrounding the lesion 14 and 42 days after stoke [58]. Mobilization of this endogenous cell pool reduces stroke damage. Blockade of NPC proliferation by infusion of cytosine arabinoside (Ara-C) for the first 7 days after brain ischemia enlarged the infarct volume and exacerbated postischemic neurological deficits. Moreover, if BDNF and VEGF were inactivated, NSCs cultured in the ischemic brain conditioned media lost neuroprotective efficacy [59]. Transplantation of human bone marrow stem cells (BMSCs) in rats after MCAO resulted in elevated BDNF, NT-3, and VEGF, enhanced endogenous NPC proliferation in both the SVZ and hippocampal subgranular zone (SGZ), and promoted NPC migration to the ischemic boundary [60].

### 2.6. Endogenous Signaling Pathways for Neurotrophin Induction

BDNF is widely expressed throughout the CNS and by most cell types, including neurons, astrocytes, microglia, and vascular endothelial cells. The classical induction mechanism involves calcium influx and phosphoactivation of the transcription factor CREB. CREB can be activated by factors released by the ischemic brain, thereby coupling BDNF synthesis and release to metabolic status. The phosphodiesterase inhibitor cilostazol increased the number of doublecortin-positive NPCs expressing phosphoactivated CREB (pCREB) and BDNF-expressing astrocytes in the ipsilateral SVZ and peri-infarct area [61]. Bone-marrow-derived stem cells cultured in ischemic brain-conditioned medium for 12 h—but not BMCs cultured in media from normoxic brain—were transformed from polygonal to fibroblast-like. This morphological change was associated with induction of 7 neurotrophic and growth factor genes and 5 receptor genes—that is, FGF-2, ILG-1, VEGF A, NGFb, and BDNF—and epidermal growth factor [62].

Therefore, many factors released from the ischemic brain may be compensatory protective signals that could potentially be exploited for neuroprotection. Epithelial cells release neurotrophic factors, including BDNF, in response to these factors. Conditioned media from cerebral endothelial cells protected neurons against oxygen-glucose deprivation* in vitro*, and this effect was blocked when condition medium was treated with free TrkB-Fc [63]. Human MSCs cultured in ischemic rat brain extract also exhibited increased production of BDNF, VEGF, and hepatocyte growth factor [64]. Stimulated microglial cells are also a source of BDNF; downregulation of the microglial response to ischemia by inhibiting the poly(ADP-ribose) polymerase-1 (PARP-1) pathway reduced BDNF production as well as markers of synaptogenesis [65]. Moreover, transplantation of a human microglial cell line—HMO6—48 hours after MCAO was found to reduce infarct volume and the number of apoptotic cells in the penumbra, while also reducing the number of host microglia/macrophages and reactive astrocytes. Transplanted HMO6 microglia also exhibited elevated GDNF, BDNF, VEGF, BMP7, and anti-inflammatory cytokines [66].

### 2.7. Summary

Enhanced neurotrophin expression in the CNS is broadly neuroprotective against acute neurological insults, including ischemia and hemorrhage. Moreover, there are a plethora of ways to increase brain neurotrophin activity, including direct infusion, transplantation of cells that express neurotrophins when exposed to the CNS environment or that are engineered to overexpress neurotrophins constitutively, small molecule mimetics, and neurotrophin inducers. The latter category of molecules presents particularly promising targets for future research, both to enhance basal ischemic tolerance and to aid in recovery following transient ischemia. Extracellular signals released by the ischemic brain also enhance neurotrophin expression. These as yet unidentified signaling pathways may also be exploited for neuroprotection. It should be noted, however, that neuroplastic changes associated with acute and chronic diseases may reduce the efficacy of these treatments (see Section 3.2).

## 3. Neurotrophins for Treatment of Chronic Neurodegenerative Diseases

There is strong evidence that many of the pathogenic mechanisms implicated in acute neurodegenerative diseases, such as oxidative stress and inflammation, are equally involved in the slower neuronal loss characteristic of chronic neurodegenerative diseases such as Alzheimer's disease (AD), Parkinson's disease (PD), various tauopathies, and Huntington's disease (HD). Therefore, the therapeutic promise of neurotrophins for the treatment of chronic neurodegenerative diseases is based in part on studies demonstrating suppression of these basic pathogenic mechanisms. Indeed, exogenous application of neurotrophins protects against neurodegeneration in animal models of AD, PD, and HD. A multitude of studies have also demonstrated that expression of neurotrophins and/or Trk receptors is reduced in the afflicted brain regions of patients and animal models. In fact, this reduction may be observed prior to symptom expression, suggesting that insufficient trophic activity may initiate or accelerate disease progression. Moreover, neurotrophins often restore or improve function in animal models in the absence of observable neuroprotection, indicating that these agents or mimetics may be therapeutically useful even in patients with advanced disease.

### 3.1. Therapeutic Efficacy of NTs, Mimetics, and Inducers in Animal Models of Chronic Neurodegenerative Disease

Animal models of chronic neurodegenerative diseases have demonstrated the therapeutic efficacy of neurotrophins, particularly BDNF. Alzheimer's disease, the most common form of age-related dementia, is characterized by accumulation of extracellular amyloid plaques of beta-amyloids (A*β*) and intracellular neurofibrillary tangles containing hyperphosphorylated variants of the cytoskeleton-associated protein tau. BDNF was found to protect cultured neurons against the cytotoxic A*β* fragments A*β*(1–42) and A*β*(25–35), and this protection was inhibited by the TrkB inhibitor K252a [67]. One of the most comprehensive studies on the protective efficacy of BDNF in animal models of AD found that injection of a lentivirus vector encoding BDNF into the entorhinal cortex (EC) improved spatial and fear memory in J20 transgenic mice expressing the mutant human amyloid precursor protein (APP), rescued EC neurons in monkeys subjected to perforant path lesions, and improved learning in delayed matching object-and-location task in aged monkeys. In this same study, direct infusion of BDNF into the EC also improved spatial memory in aged rats [68]. Infection with a viral vector encoding BDNF was found to enhance BDNF expression and improve memory deficits in the Tg2576 transgenic mouse model of AD. Significantly, the vector used—Sendai virus (SeV)—is highly infective but does not integrate into the host genome and is nonpathogenic in humans. Thus, SeV vectors hold great potential for the safe delivery of neuroprotective peptides to the brain [69]. In addition to neuroprotection, BDNF may slow disease progression by inhibiting amyloid production. BDNF was found to induce the expression of sorting protein-related receptor with A-type repeats (SORLA), a protein that reduces APP processing and plaque formation, and no longer reduce A*β* in SORLA-deficient mice [70].

In aged 3xTg-AD mice, a model exhibiting amyloid plaques, neurofibrillary tangles, and age-associated cognitive decline, the injection of NSCs into the bilateral hippocampus improved spatial memory in the MWM but did not reduce amyloid or tau pathology. Preservation of cognitive function effect was absent when BDNF expression was knocked down using a targeted shRNA [71]. The fact that BDNF can improve cognitive function without reducing neuropathology suggests that this strategy may be useful in advanced AD patients.

Cognitive dysfunction in AD arises in part from the loss of septal cholinergic neurons that are project to the hippocampus. Toxic A*β*(1–42) induced the death of septal neurons* in vitro*, and this effect was reduced by BDNF (as well as by IGF-1 and GDNF) and by coculture of septal neurons with NSCs overexpressing neurotrophins [72]. The cholinergic neurotoxin (192) IgG-saporin reduced both MWM performance in rats and BDNF expression, while the reversal of these effects by the clinical cholinesterase inhibitor galantamine was associated with enhanced hippocampal BDNF levels [73].

Transgenic mice overexpressing APP and infected with a lentiviral BDNF vector at 2 months of age showed significantly reduced neurodegeneration in the EC and improved fear memory at 7 months of age [74], indicating that these therapeutic effects are relatively long-lasting. BDNF may also preserve neuronal viability and function in AD and other tauopathies by reducing tau phosphorylation. Indeed, BDNF reduced phosphorylation at multiple sites, an effect blocked by inhibition of TrkB, PI-3 kinase, and GSK 3*β* [75]. The TrkB agonist 7,8-dihydroxyflavone rescued deficient spatial working memory in 5XFAD mice and reduced expression levels of *β*-secretase (BACE1), A*β*(1–40), and A*β*(1–42) [76].

In accord with the aforementioned results on neuroprotective strategies against stroke-induced neuronal damage, many potentially effective treatments for AD may be mediated by enhanced BDNF signaling. A curcumin derivative, J147, increased BDNF expression and reduced A*β*(1–42) and A*β*(1–40) in aged AD mice (APP/swePS1ΔE9). Furthermore, these changes were associated with enhanced long-term potentiation, a putative neurocellular mechanism for associative learning, and a reduction in oxidative stress [77]. In another study, J147 improved both spatial and fear memory in aged AD mice and upregulated NGF, BDNF, and several synaptic proteins induced by BDNF, including homer-1, a protein critical for the trafficking of glutamate receptors. Moreover, J147 was shown to be nontoxic at millimolar serum concentrations while demonstrating neuroprotection in nanomolar doses* in vitro* [78]. Elevated A*β* reduced the colocalization of the synaptic marker synaptophysin with AMPA receptor subunits GluR1 and GluR4. As trafficking of AMPA receptors underlies forms of synaptic plasticity linked to learning, this disruption may explain the learning deficits associated with AD. Furthermore, exogenous BDNF was found to restore synaptic AMPA receptor trafficking [79].

Sildenafil (Viagra) also rescued cognitive function in the AD model mice, but without reducing the amyloid burden [80]. The neuroprotective effects of NPY* in vitro *against toxic A*β* fragments were also found to be associated with neurotrophin upregulation [81]. The antidepressant amitriptyline enhanced BDNF and TrkB expression in the hippocampus, promoted neurogenesis, and improved spatial and object recognition memory in aged 3xTg mice [82]. The multiple sclerosis drug fingolimod phosphate (a sphingosine-1-phosphate receptor agonist) enhanced BDNF expression and protected mouse cortical neurons against A*β* toxicity, an effect blocked by inhibition of BDNF-TrkB-ERK signaling [83]. Neuropep-1 is a new BDNF inducer that rescued spatial memory deficits in 3xTg-AD mice. In addition, Neuropep-1 also reduced the A*β* burden, possibly by enhancing expression of the insulin/A*β*-degrading enzyme [84]. Sodium phenylbutyrate (NaPB) induced BDNF and NT-3 expression in astrocytes by activating the PKC-CREB signaling pathway and improved spatial learning and memory in a mouse model of AD [85].

Significant neuroprotective effects of neurotrophins, particularly BDNF, have also been demonstrated in animal models of PD. Intrathecal infusion of BDNF suppressed Parkinsonian signs in monkeys one week after treatment with the mitochondrial toxin 1-methyl-4-phenyl-1,2,3,6-tetrahydropyridine (MPTP), which selectively destroys dopaminergic neurons and is widely used to model PD pathology. Moreover, BDNF infusion reduced neuronal loss in the SN and the extent of rescue was correlated with the preservation of function [86]. Transplantation of astrocytes expressing tyrosine hydroxylase (TH) and/or BDNF blocked 6-OHDA-induced degeneration and the effect of TH plus BDNF was more effective than either alone [87]. Injection of a BDNF viral vector did not increase TH-positive SN neurons six months after unilateral 6-hydroxydopamine (6-OHDA) injection but did block amphetamine-induced ipsilateral rotation [88], a PD-like behavior. Transplantation of rat fibroblasts secreting human BDNF near the SN prior to striatal MPP+ administration enhanced neuronal survival compared to transplantation of wild-type fibroblasts [89].

Other PD treatment strategies are associated with BDNF induction. Repetitive transcranial magnetic stimulation (rTMS) rescued DA neurons, improved locomotor function, and induced expression of BDNF, GDNF, PDNF, and VEGF in the 6-OHDA model of PD [90]. Similar to results in stroke models, prior exercise reduced Parkinsonian behavior (apomorphine-induced rotations), rescued dopaminergic neurons, and reversed the early reduction in BDNF expression observed following PD model induction. Neuronal rescue was reduced by inhibition of BDNF signaling [91]. Prior exercise (4 weeks) also reduced the loss of dopaminergic neurons and improved motor function following intraperitoneal injection of the activator of inflammation lipopolysaccharide (LPS), an effect blocked by a TrkB antagonist and mimicked by intracerebral perfusion of BDNF [92]. Successful motor rehabilitation also increased serum BDNF in PD patients [93]. The therapeutic efficacy of deep brain stimulation (DBS) targeting the subthalamic nucleus (STN-DBS), a widely used PD treatment, may also depend on BDNF induction; STN-DBS increased BDNF but not GDNF expression in the SN, striatum, motor cortex (M1), and hippocampus—an effect that was blocked by an NMDA receptor antagonist [94]. A diet high in omega-3 polyunsaturated fatty acids (n-3 PUFAs) also protected against MPTP-induced death of dopaminergic neurons and induced striatal expression of BDNF [95]. Pretreatment with the ampakine and BDNF inducer CX614 significantly reduced MPP(+)-induced toxicity in brain slices, an effect blocked by an inhibitor of TrkB [96]. Another novel neurotrophin inducer, PYM50028, also reduced the loss of SN dopaminergic neurons in mice when administered after MPTP [97].

### 3.2. Is Dysregulation of Neurotrophin Involved in the Pathogenesis of Chronic Neurodegenerative Disease?

Deficient neurotrophin signaling has also been implicated in neurodegenerative disease progression. A*β* reduction in neurotrophin expression in vulnerable brain regions has been widely reported, while genetic association studies have identified specific neurotrophin gene alleles as risk factors [98]. As discussed below, however, many of these studies also indicate that pathogenic processes may interfere with neurotrophin signaling and thereby reduce the effects of exogenous neurotrophin administration.

Expression levels of neurotrophins and Trk receptors are altered in neurodegenerative diseases, but the functional implications are currently unclear. The large-scale Framingham Study found a specific association between low-serum BDNF and dementia/AD risk in elderly women [99]. Reduced BDNF levels were also observed in the cortex of female APdE8 AD model mice [100]. A comparison of several transgenic AD model mice revealed that cortical BDNF mRNA expression was reduced in two models—namely, APP(NLh) and TgCRND8—compared to wild types, and all models studies exhibited an inverse correlation between toxic A*β* levels and BDNF expression [101]. In aged model mice, however, BDNF immunoreactivity was elevated around amyloid plaques [100]. It was suggested that this elevation could act to sequester BDNF and reduce BDNF-TrkB signaling. Alternatively, it may represent a reactive response to damage.* In situ* hybridization also showed a several-fold increase in BDNF mRNA around amyloid plaques due to elevated microglial and astrocytic expression [102].

Despite uncertainties concerning brain neurotrophin levels during the different phases of neurodegenerative disorders, there is substantial evidence that neurotrophin signaling is disrupted in AD and PD. For instance, A*β* can also alter the expression of TrkB isoforms [103]. In cultured neurons, sublethal doses of A*β* inhibited BDNF-MAPK/ERK and BDNF-PI3-K signaling by interfering with insulin receptor substrate-1 and Shc, two docking proteins that link TrkB to the activation of downstream kinase cascades [104]. In addition, A*β* treatment increased truncated TrkB.T1 and decreased full-length TrkB.TK expression in cultured neurons; furthermore, overexpression of TrkB.T1 in APdE9 mice enhanced cognitive deficits [105]. The AD-linked gene presenilin 1 can also regulate surface expression of TrkB [106]. Amyloid-*β* accumulation also interfered with CREB activation, while viral delivery of CREB-binding protein (CBP) restored CREB function and improved memory deficits in an AD model. These changes were associated with elevated BDNF but no reduction in A*β* or tau pathology [107]. Finally, the Tg2576 mouse exhibited reduced retrograde axonal transport of BDNF that was mitigated by inhibition of *γ*-secretase, implicating A*β* accumulation in reduced BDNF-nuclear signaling. Moreover, the administration of A*β* alone reduced retrograde transport [108]. This deficit may stem from deficient expression of ubiquitin C-terminal hydrolase L1 (UCH-L1), a deubiquitinating enzyme reduced in AD, as a UCH-IL inhibitor also reduced retrograde transport of BDNF [109]. Inhibition of BDNF signaling independent of BDNF expression levels may be a major impediment to the clinical application of techniques that only augment endogenous BDNF or TrkB.

This disruption of neurotrophin signaling could further exacerbate neuronal injury. AD patients expressing the Met allele of BDNF showed enhanced atrophy in several brain regions, including anterior cingulate cortex, posterior cingulate cortex, and precuneus, compared to Val/Val carriers [110]. Several SNPs in the BDNF gene were associated with more rapid brain atrophy over two years [111]. Carriers of both the BDNF Met allele and the AD risk factor APOE *ε*4 exhibited enhanced degeneration in the precuneus, orbitofrontal cortex, gyrus rectus, and lateral prefrontal cortex, as well as episodic memory deficits that were correlated with amyloid burden [112]. Moreover, a meta-analysis of studies on the BDNF 196A/G polymorphism and AD risk reported a significant association in females [113]. Carriers of the Met allele also showed faster cognitive decline and greater brain atrophy over 36 months compared to Val/Val carriers; however, this only occurred in elderly adults with high basal A*β* at the start of the study, suggesting that neurotrophin dysregulation accelerates rather than initiates neurodegeneration in AD [114].

Deficient BDNF signaling is also implicated in PD and HD progression. In the substantia nigra of PD patients, BDNF expression is reduced, even in surviving neurons [115, 116]. BDNF is also reduced in Huntington's disease, and BDNF expression is necessary for the survival of medium-sized spiny striatal neurons lost during HD progression [117]. Continuous infusion of a BDNF antisense oligonucleotide into the SN of rats induced Parkinsonian behaviors, including apomorphine-evoked rotation, and resulted in the loss of TH-positive neurons and dopamine uptake [118]. Mice lacking BDNF expression in the mid- and hindbrain demonstrated motor impairments and reduced numbers of TH-positive neurons, while no reduction was observed in the calbindin- and calretinin-positive neurons spared in PD [119]. Exercise mitigated Parkinsonian signs, improved mitochondrial function, and elevated BDNF/GDNF expression in a mouse PD model [120]. Reduced TrkB expression reduced the number of SN neurons in elderly mice (21–23 months old) and resulted in accumulation of alpha-synuclein in surviving neurons [121]. Aged TrkB hypomorphic mice were hypersensitive to MPTP and showed enhanced age-related loss of dopaminergic neurons in the SN [122]. Finally, nigral dopaminergic neurons expressing TrkB were more resistant to MPTP than neurons expressing mainly TrkC [123].

## 4. Conclusions and Future Directions

These studies indicate that neurotrophins can protect neurons against a variety of pathological insults, including ischemia and neurotoxins such as A*β*. Moreover, a variety of innovative methods have been developed to noninvasively enhance neurotrophin expression in the brain. Despite these advances, neither neurotrophin delivery nor induction is used in the clinic as a routine treatment. All of these disorders are associated with multiple pathogenic processes, so it is possible that effective treatment will require multiple complementary strategies. Furthermore, these disorders appear to disrupt neurotrophin signaling, so future work should concentrate on the targeted delivery of neurotrophins to vulnerable tissues and methods of preserving neurotrophin function in the diseased brain.

## Abbreviations

AD: Alzheimer's disease
PD: Parkinson's disease
HD: Huntington's disease
NGF: Nerve growth factor
BDNF: Brain-derived neurotrophic factor
CNS: Central nerve system
BBB: Blood brain barrier
Trk: Neurotrophin receptor
MCAO: Middle cerebral artery occlusion
GDNF: Glial-derived neurotrophic factor
IGF-1: Insulin-like growth factor-1
VEGF: Vascular endothelial growth factor
NSC: Neural stem cell
MSC: Mesenchymal stem cell
BM: Bone marrow
IBZ: Ischemic boundary zone
MWM: Morris water maze
NPC: Neural progenitor cells
CP: Choroid plexus
SN: Substantia nigra
NMDA: N-methyl-D-asparate
FGF-2: Fibroblast growth factor-2
SVZ: Subventricular zone
NOS: Nitric oxide synthase
HELP: High-voltage electric potential
RCI: Respiratory control index
SGZ: Subgranular zone
PARP-1: poly(ADP-ribose) polymerase-1
A*β*: Beta-amyloids
APP: Amyloid precursor protein
EC: Entorhinal cortex
MPTP: 1-Methyl-4-phenyl-1,2,3,6-tetrahydropyridine
TH: Tyrosine hydroxylase
LPS: Lipopolysaccharide.
